# Mutational characterization of HBV reverse transcriptase gene and the genotype-phenotype correlation of antiviral resistance among Chinese chronic hepatitis B patients

**DOI:** 10.1080/22221751.2020.1835446

**Published:** 2020-10-30

**Authors:** Ya Fu, Songhang Wu, Yuhai Hu, Tianbin Chen, Yongbin Zeng, Can Liu, Qishui Ou

**Affiliations:** a Department of Laboratory Medicine, The First Affiliated Hospital, Fujian Medical University, Fuzhou, China; b Clinical Laboratory Diagnostics, The First Clinical College, Fujian Medical University, Fuzhou, China; c Fujian Key Laboratory Medicine, The First Affiliated Hospital, Fujian Medical University, Fuzhou, China; d Gene Diagnosis Research Center, Fujian Medical University, Fuzhou, China; e Department of Hepatobiliary Surgery, The First Affiliated Hospital, Fujian Medical University, Fuzhou, China

**Keywords:** Hepatitis B virus, reverse transcriptase, resistant mutation, genotype, phenotype

## Abstract

**Background and Aims:** The drug resistance of hepatitis B virus (HBV) originates from mutations within HBV reverse transcriptase (RT) region during the prolonged antiviral therapy. So far, the characteristics of how these mutations distribute and evolve in the process of therapy have not been clarified yet. Thus we aimed to investigate these characteristics and discuss their contributing factors.

**Methods:** HBV RT region was direct-sequenced in 285 treatment-naive and 214 post-treatment patients. Mutational frequency and Shannon entropy were calculated to identify the specific mutations differing between genotypes or treatment status. A typical putative resistance mutation rtL229V was further studied using *in-vitro* susceptibility assays and molecular modeling.

**Results:** The classical resistance mutations were rarely detected among treatment-naive individuals, while the putative resistance mutations were observed at 8 AA sites. rtV191I and rtA181T/V were the only resistance mutations identified as genotype-specific mutation. Selective pressure of drug usage not only contributed to the classical resistance mutations, but also induced the changes at a putative resistance mutation site rt229. rtL229V was the major substitution at the site of rt229. It contributed to the most potent suppression of viral replication and reduced the *in-vitro* drug susceptibility to entecavir (ETV) when coexisting with rtM204V, consistent with the hypothesis based on the molecular modeling and clinical data analysis.

**Conclusions:** The analysis of mutations in RT region under the different circumstances of genotypes and therapy status might pave the way for a better understanding of resistance evolution, thus providing the basis for a rational administration of antiviral therapy.

## Introduction

Nearly 240 million people worldwide suffer from the chronic infection of hepatitis B virus (HBV) and its consequent progression such as cirrhosis and hepatocellular carcinoma [1,2]. Several nucleoside/nucleotide analogues (NAs) which inhibit HBV reverse transcriptase (RT) have been developed to optimize the antiviral therapy. Available NAs in clinical use include Lamivudine (LMV), adefovir (ADV), telbivudine (LdT), entecavir (ETV), and tenofovir (TDF). Prolonged use of these antiviral compounds will contribute to the constant elimination of HBV, also along with the increasing emergence of resistance mutants that may markedly reduce the initial effects of these treatments.

It has become clear that viral resistance originates from mutations in the nucleotide binding motif of HBV RT region [3]. Phenotypic studies showed that the LMV resistance arising from rtM204V/I in the YMDD motif, could negate sensitivity to LMV by more than 100-fold decrease compared with wild-type [4]. As compensatory changes, some other additional mutations such as rtL180M, rtV173L can also be included when the LMV resistance is occurring. ETV appears less likely to develop drug resistance due to its property of high barrier to resistance, which makes it be recommended as the first-line antiviral regimen at present. The classic ETV resistance mutations rtT184G/rtS202I/rtM250V will reduce susceptibility to ETV, only in conjunction with additional LMV resistance mutations, such as rtM204V/I [5].

Lack of a proofreading function of HBV reverse transcriptase leads to an error-prone replication of HBV. As a result, the HBV evolutionary dynamics frequently yields a high rate of mutations, thus producing genetically heterogenous viral populations. These variants, commonly with altered fitness, can even be pre-existing in a treatment-naive patient, and be selected as dominant strains due to the drug pressure or host immunity change [6]. This phenomenon has already been confirmed as a main factor explaining how antiviral resistance occurs and persists during prolonged NAs therapy [7]. A number of studies have indicated the substantial role of the YMDD motif and other classical antiviral resistance mutations in the post-treatment patients, but most of them have not yet reached an agreement over how these mutations distribute in treatment-naive patients [8–11]. Besides, large amount of mutations as natural genome variability are mainly characterized as either pre-treatment mutations or putative resistance mutations that might be selected under drug pressure, yet reported without sufficient evidence in vitro to classify the functional relevancy [12–14]. To scrutinize the presence of these mutations in different genotypes and treatment status will be beneficial to understand their role in the process of resistance evolution, and even to preliminarily explain why the risk of resistance varies between individuals.

Therefore, this study aimed to characterize mutations across HBV RT sequences in 499 chronic hepatitis B (CHB) patients in China. We expected to explore the mutational distribution and frequency in the presence/absence of selective drug pressure, as well as in different genotypes of HBV by Sanger sequencing. Furthermore, we performed *in-vitro* susceptibility assays to verify the resistance efficacy of the mutation rtL229 V, a potential ETV-resistance site found in the study.

## Methods

### Patients

The study was approved by the Ethics Committee of the First Affiliated Hospital of Fujian Medical University. Informed consent on entry into the trials was obtained from all patients. A total of 499 HBV-infected patients including 285 treatment-naive individuals and 214 post-treatment individuals were enrolled at the First Affiliated Hospital of Fujian Medical University (Fujian, China) from Jan 2011 to Dec 2016. The inclusion criteria were 18–65 years of age, presence of hepatitis B surface antigen (HBsAg) and HBV DNA at least 6 months prior to treatment. Patients co-infected with the hepatitis A/C/D virus, human immunodeficiency virus, autoimmune liver disease, primary biliary cirrhosis, and alcohol or drug abuse were excluded. Treatment-naive patients were defined as those who have no history of receiving any NAs therapy. For the post-treatment patients, the median duration for antiviral therapy (LMV/ADV/LdT/ETV) was 64 (12–108) weeks before their samples were detected in our study. All the serum samples were collected and stored at −80°C.

### Diagnostic tests

Serum alanine aminotransferase (ALT) and aspartate aminotransferase (AST) were determined by automated biochemical technique (Siemens Healthcare Diagnostics, USA) with cutoff value of 40 IU/L. Serum HBsAg, anti-HBs, hepatitis B e antigen (HBeAg), anti-HBe and anti-HBc were measured on the wholly automatic immune fluorescence analyzer Abbott Type i4000 (Abbott Laboratories, USA) using the original, corollary commercial kits. Serum HBV DNA was quantiﬁed using the TaqMan polymerase chain reaction (PCR) assay (Sansure Biotech, China) on Roche LightCycler 480 Real-Time PCR System (Roche, Switzerland) with a low limit of quantification of 500 IU/ml.

### HBV DNA extraction and Sanger sequencing

HBV DNA was extracted from 500 μL patient serum according to the manufacturer’s instructions of the TIANamp Genomic DNA Kit (Tiangen Biotech, Beijing). DNA samples were eluted with 100 μL sterilized water. The HBV RT gene was amplified by PCR using forward primer 5-CTCATGTTGCTGTACAAAACC-3 (nt559-nt579) and reverse primer 5-CAATTCKTTGACATACTTTCCA-3 (nt1000-nt979). PCR was performed in a 50μL mixture containing 2.5 mM MgCl2, 400μM dNTP and 2.5U of Taq polymerase (TAKARA, Japan). PCR conditions were as follows: 94°C for 5 min; 35 cycles of 94°C for 1 min, 60°C for 1 min and 72°C for 1 min; then 72°C for 10 min. The PCR products were purified using a QIAquick Gel Extraction Kit (Qiagen, Germany) and directly sequenced (The Beijing Genomics Institute, Shenzhen, China).

### HBV genotyping and mutation analysis

HBV genotyping was performed by phylogenetic analysis using NCBI Viral Genotyping Tool: www.ncbi.nlm.nih.gov/projects/genotyping/formpage.cgi, together with 17 B-genotype DNA sequences (AY217364, AY217358, AY206387, AY206383, AY206380, AY206375, AY206373, FJ518812, FJ518811, FJ386688, FJ386684, FJ386683, FJ386682, FJ386681, FJ386680, FJ386676 and FJ386675) and 17 C-genotype DNA sequences (EU916228, AY217372, EU439025, EU439012, EU916232, EU916229, EU916227, EU916226, EU916225, EU916224, EU916223, EU916222, EU916221, EU916220, EU916219, EU916218 and EU916217) from Genbank.

The mutations were analyzed by comparing the RT gene sequences with genotype-matched HBV consensus sequence generated from the sequences obtained in this study and the HBV wild-type sequences previously published in literature. The categories of potential NAs resistance mutation (NA-r mutation) were classified into four categories: 1-primary, 2-secondary, 3-putative, and 4-pre-treatment mutations, as recommended from a previous report by Liu et al 2010 [14]. Classical NAs resistance mutation (NA-r mutation) denotes the mutational categories of primary and secondary mutation, which displays a certain efficacy on reduced susceptibility to NAs. Shannon entropy (Hn) is an important parameter determining if there is greater variability in one point relative to the consensus sequence, and was calculated at nucleotide and amino acid (AA) levels using the online tool at the Los Alamos National Laboratory: http://www.hiv.lanl.gov/content/sequence/ENTROPY/entropy_one.html. The formula for calculation is *Hn* = $-\sum_{i=1}^{n}(p\text{i ln}p\text{i) }$, where *pi* represents the frequency of each nucleotide or AA type at the specific site and *n* represents the total number of nucleotide or AA types.

### In-silico prediction of HBV RT three-dimensional structure

To confirm more functional relevancy with regard to rtL229 substitutions, we built three-dimensional model of wild-type and mutated HBV RT by the online I-TASSER server. This modeling program as established by Zhang et al. (2015) [15] could be used at the following website: https://zhanglab.ccmb.med.umich.edu/I-TASSER/. The final structures were used for the docking simulations. The binding affinity of HBV RT to various antiviral agents, including ETV-TP (entecavir triphosphate) and LMV-TP (lamivudine triphosphate), were evaluated by the docking program from myPresto system reported by Morikami et al. [16]. Each model of Wild-type or mutated RT was subsequently compared by evaluating its three-dimensional structure as well as binding energy.

### Cell culture and site-directed mutagenesis for generating wild-type and mutated plasmid

Plasmid expression vector p1.2DNA encoding a wild-type 1.2 × unit length HBV genome (B-genotype) ensures sufficient HBV DNA replication to meet the requirement for detection of phenotypic resistance. Three different mutations, rtM204V, rtL229V and rtM204V + rtL229V were introducing into p1.2DNA by site-directed mutagenesis (TransGen Biotech, Beijing, China). All artificial mutant constructs were subsequently sequenced to confirm that no additional mutations had been introduced during the site-directed mutagenesis process.

Bel-7404 human hepatoma cells were cultured in Dulbecco’s modified Eagle’s medium (DMEM)(Gibco, USA), supplemented with 10% fetal bovine serum(Gibco, USA), penicillin (100 U/mL) and streptomycin (100 μg/mL) at 37°C in a humidified 5% CO_2_ incubator.

### Phenotypic resistance assays for wild-type HBV and mutants

Wild-type or mutated HBV genomes were transiently transfected into Bel-7404 cells as follows. Cells were seeded in six-well plates at a density of 8 × 10 ^5^ cells/well 24 h earlier and were transfected with 4 μg plasmid DNA and 10 μl Lipofectamine 2000 (Invitrogen, USA) according to the manufacturer’s protocol. Drug was added into the culture medium 6 h after transfection and included for 6 days. The medium was changed every 2 days, and fresh drug-free or drug-containing medium was added. The concentrations of LMV, ETV, and TDF for the assays were 0, 0.01, 0.1, 1, 10 and 100 μM (LMV), 0, 0.005, 0.025, 0.125 and 0.625 μM (ETV), 0, 0.25, 0.5, 1, 2 and 4 μM (TDF). After 6 days of drug treatment, the cell culture supernatant in each well was collected and detected of HBsAg, HBeAg, HBV DNA levels. All these biomarkers were separately assayed using the method as mentioned above. For each drug, the concentration inhibiting by 50% the amount of supernatant viral DNA (IC_50_) was calculated.

### Statistical analysis

Differences in measurement data were evaluated by *T*-test, Mann–Whitney U test, one-way analysis of variance and multiple comparisons with IBM SPSS Statistics software (Version 22.0.0; IBM, Armonk, New York, USA). All *P*-values were two-tailed. *P* < 0.05 was considered statistically significant.

## Results

### Patient characteristics

Multiple comparisons of the patients’ characteristics are shown in Table 1. HBV RT gene was sequenced by Sanger sequencing in 285 treatment-naive patients and 214 post-treatment patients with the genotype ratio of 56.8% (162/285) and 52.8% (113/214) B-genotype respectively in each group (*P* = 0.369). This data basically reflected the similar genetic background of HBV isolates between each of the patient group, and coincided with the prevalent characteristic of the HBV genotype in southern China [14]. Significant differences were found in gender, age, HBsAg, Anti-HBc, AST and HBV DNA among the two treatment groups. All the patients in post-treatment group were treated using NAs mono-therapy (199/214) or combination therapy (15/214).

**Table 1.** Characteristics of 499 patients enrolled in this study.

	Treatment-naive (*N* = 285)	Post-treatment (*N* = 214)	***χ^2^/t****/Z*	***P***
Gender (male/female)*	204/81	177/37	8.388	0.004
Genotype (B/C)*	162/123	113/101	0.806	0.369
HBeAg (positive/negative)*	184/101	139/75	0.008	0.928
**Age (year), mean ± SD^&^**	35.785 ± 13.548	41.533 ± 13.596	−4.683	<0.001
**HBV DNA (log_10_ IU/mL), mean ± SD^&^**	6.203 ± 1.513	5.862 ± 1.418	2.564	0.011
**Anti-HBcAg (S/CO), mean ± SD^&^**	12.541 ± 3.388	11.632 ± 2.465	3.321	0.001
**HBsAg (IU/mL), median (IQR)^#^**	4715.000 (1918.445∼19205.010)	3525.000 (1265.920∼14032.430)	−2.101	0.036
**ALT (U/L), median (IQR)^#^**	166.000 (60.000∼404.000)	210.500 (64.250∼530.750)	−1.628	0.104
**AST (U/L), median (IQR)^#^**	83.500 (45.000∼182.000)	132.500 (48.00∼275.750)	−2.838	0.005
**AFP (ng/mL), median (IQR)^#^**	8.095 (2.748∼35.125)	6.260 (2.553∼30.948)	−1.243	0.214
**Drug usage**	–	LMV, 70; ADV, 55; LdT, 27; ETV, 47; LMV + ADV, 11; LdT + ADV, 1; LMV + ETV, 2; ETV + ADV, 1	—	–

*, differences were determined for statistical significance using the Chi-square test;

^&^ , differences were determined for statistical significance using the Student’s *t*-test ;

^#^ , differences were determined for statistical significance using the Mann-Whitney U test.

### Analysis of genotype-dependent sites

**Figure 1.**
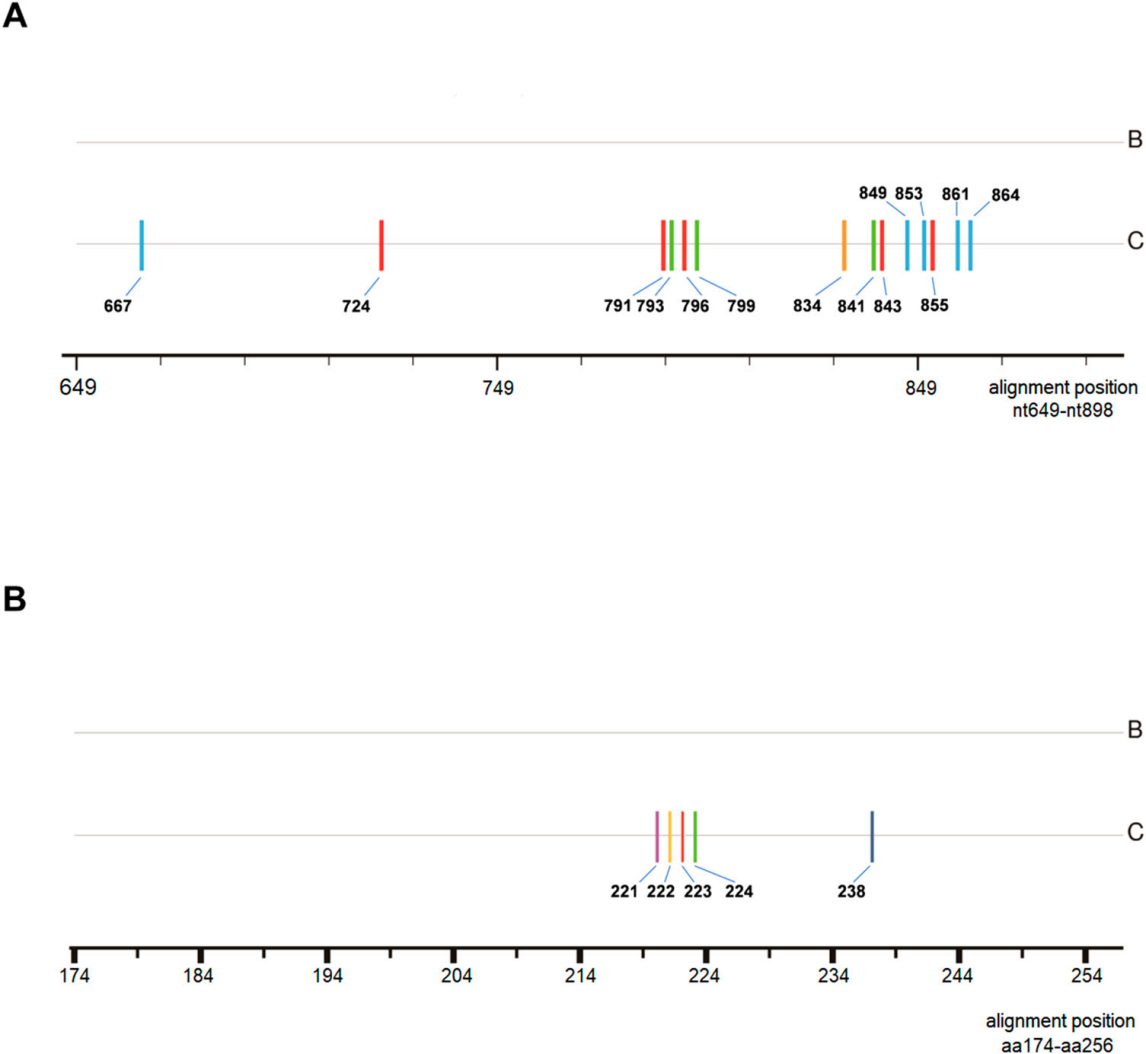
Consensus sequence alignment between B-genotype and C-genotype HBV at nucleotide and amino acid level. Sticks with different colors indicate how genotype-dependent sites distribute within RT region. (A) genotype-dependent nucleotide sites within RT. blue: cytosine; red: thymine; green: adenine; yellow: guanine; (B) genotype-dependent amino acid sites within RT. purple: phenylalanine; yellow: threonine; red: serine; green: isoleucine; dark blue: asparagine; aa: amino acid.

Comparing treatment-naive samples that were genotype B to those that were genotype C, genotype-dependent sites were verified at both nucleotide and amino acid levels (Figure 1). The results from 285 samples revealed that 14 nucleotide sites might be genotype-dependent within HBV RT region, that is, T or C at nt667 and nt864, C or T at nt724 and nt843, A or T at nt791, nt855 and nt861, G or A at nt793 and nt799, G or T at nt796,A or G at nt834, C or A at nt841, A or C at nt849 and nt853 were significantly correlated with genotype B or genotype C respectively (*P* < 0.0001) (supplementary data: Table S1). Furthermore, the presence of genotype-dependent amino acid polymorphic sites was also confirmed at 5 AA residues with genotype B and genotype C respectively containing tyrosine or phenylalanine at rt221, alanine or threonine at rt222, alanine or serine at rt223, valine or isoleucine at rt224, histidine or asparagine at rt238 (supplementary data: Table S2). Referring to the previous literature, the dominant AA at a particular site carried a substantial weight in the identification of genotype B and C, however it was noteworthy that some low frequency AA residues like histidine at rt221, threonine at rt223, leucine at rt224, could be described as spontaneously occurring mutations and had no characteristics of genotype-dependence in this study [14].

### Analysis of mutation frequency and entropy

**Figure 2.**
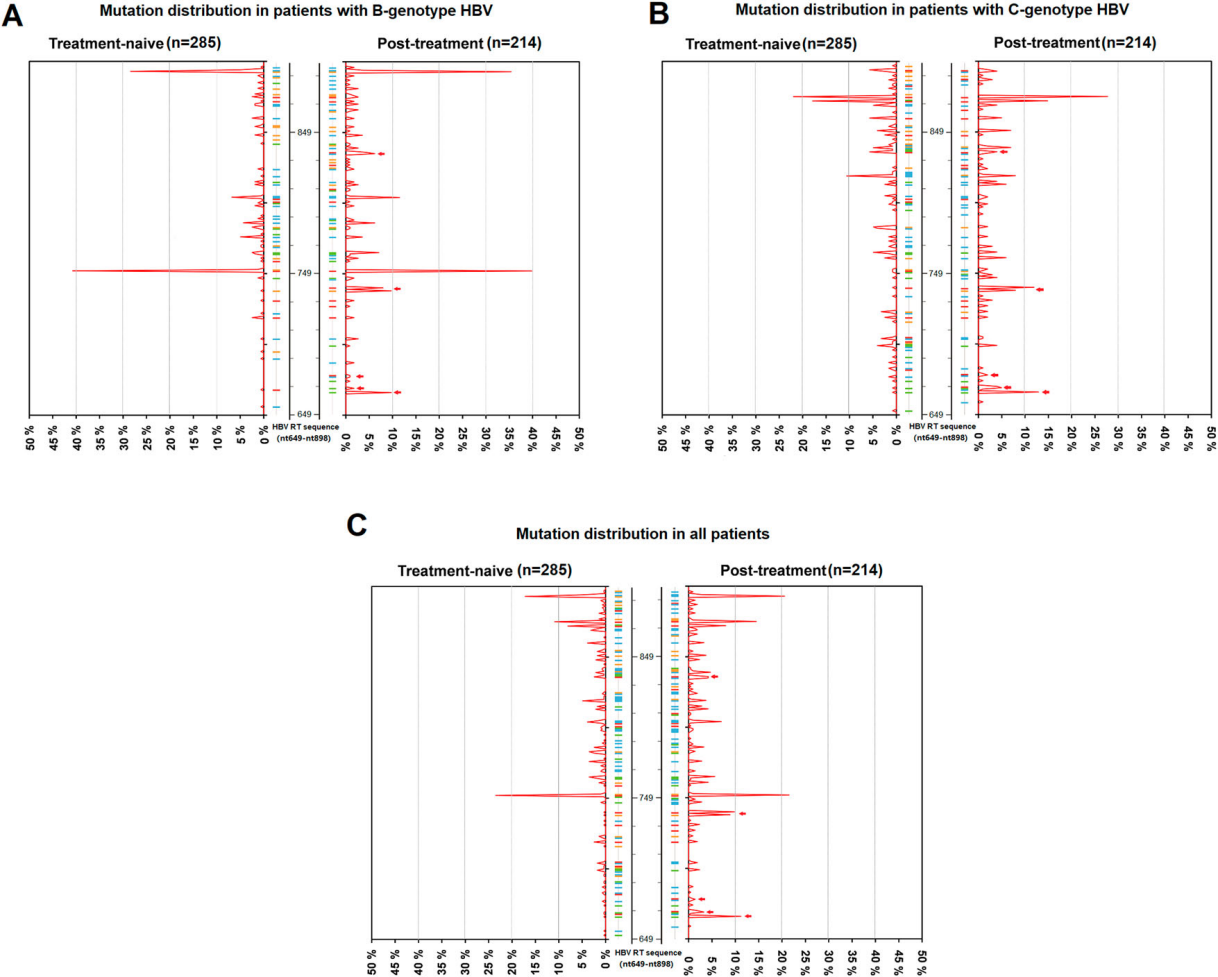
Mutation distribution in treatment-naive and post-treatment patients. 285 treatment-naive and 214 post-treatment patients were sequencing, the frequency of each sites of HBV RT region was analyzed. 250 nucleotide sites within RT region (nt649∼nt898) are displayed in the histogram. The mutated nucleotides are shown by the color sticks near the center axis (blue: cytosine; red: thymine; green: adenine; orange: guanine). Peaks reflect the frequency of mutation at nucleotide sites. Small red arrows reflect the site of primary and secondary resistance mutations, from bottom to top: rtL180M, rtA181V/T/I, rtT184S/L, rtM204V/I, rtN236T/I. (A) The frequency of mutations only in the cohort of patients with B-genotype HBV. (B) The frequency of mutations only in the cohort of patients with C-genotype HBV. (C)The frequency of mutations in the cohorts of all patients. nt: nucleotide.

Through screening the HBV RT gene from nt649 to nt898, mutations were analyzed by a comparison with the HBV consensus sequence. The frequency distribution of each mutation was shown by percentage (Figure 2). In brief, RT gene displayed a genetic variability in the terms of mutations within 4 functional domains (B–E) and 3 interdomains (B–C, C–D, D–E). Five mutations: ntA753T/G, ntC876T, ntA894G/C occurred with the rates up to more than 10% in both treatment-naive group and post-treatment group. Only 1.1% (3 of 285, rtA181T/rtM204V/rtL180M + rtM204I) of HBV isolates harboring classical primary or secondary resistance mutation were observed among treatment-naive patients. Putative resistance mutations were observed at 8 AA sites among the panel of treatment-naive patients; they were rtV191I, rtS213T, rtV214A, rtE218D, rtL229V, rtI233V, rtN238S, rtS/C256G. The classical NA-r mutations rtL180M (ntT/C667A) and rtM204V/I (ntA739G or ntG741T/A), were treatment-specific and were preferentially present in post-treatment patients. The NA-r mutation patterns and their frequencies are summarized in Table S3.

**Figure 3.**
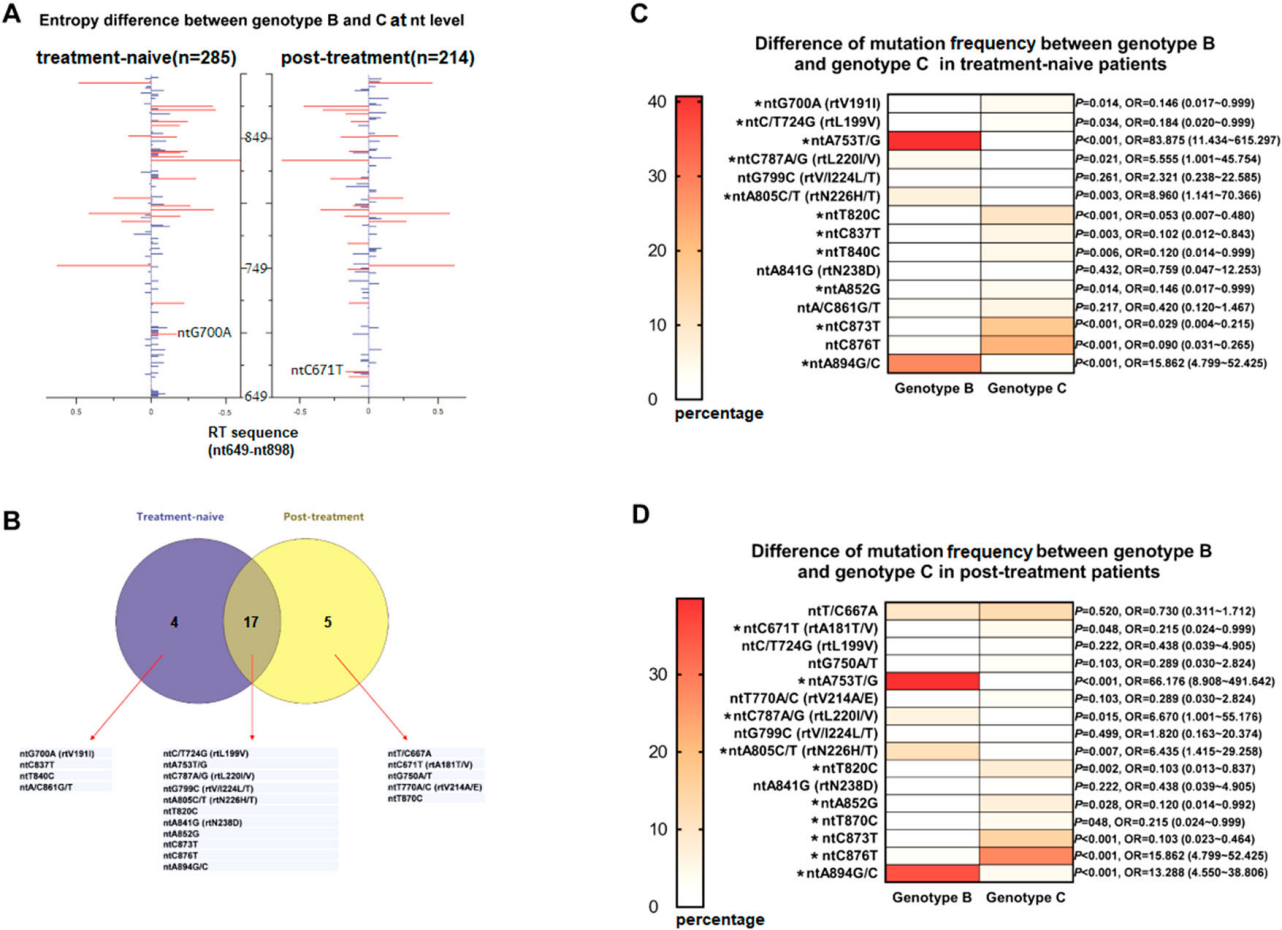
Analysis of mutations between B-genotype and C-genotype HBV in CHB patients. (A) 250 nucleotide sites within RT region (nt649-nt898) were aligned and assessed for Shannon entropy of each nucleotide sites. Bars reflect the Shannon entropy (genetic variability) at nucleotide sites. Significant differences of entropy via a pairwise comparison between genotype B and C are shown with red bars, and differences that were not statistically significant are shown in blue. (B) 20 nucleotide sites with significant differences of entropy between genotype B and C were screened, their relations with the treatment status are shown. (C-D) Frequency of mutations at the 20 sites were determined and compared between genotype B and C by *t*-test*P < 0.05. rt: reverse transcriptase; nt: nucleotide.

Genotypes B and C were compared regarding the number of isolates with mutation within the RT region. No statistical differences were observed between genotypes B and C, respectively (90/275, 32.7% vs 79/224, 35.5%, *P* = 0.57). Since Shannon entropy (Hn) can be used as a parameter to evaluate the sequence variability of HBV, we calculated Hn for each site of RT gene from nt649 to nt898 and displayed the results by a pairwise comparison between the two sets of genotypes in Figure 3(A). Remarkably, a total of 20 nucleotide sites differed significantly in Shannon entropy owing to the type of genotype, including 4 sites in treatment-naive group, 5 sites in post-treatment group and 11 sites in both (Figure 3(B)). After scrutinizing the frequency of mutations within these sites, only two NA-r mutations, ntG700A (rtV191I, *P* = 0.014) and ntC671T (rtA181T/V, *P* = 0.048) from treatment-naive and post-treatment samples, respectively, were identified as genotype-specific mutations (Figure 3(C–D)). The sequencing results also indicated some significant differences between genotype B and genotype C regarding the frequency of some other synonymous mutations. For instance, ntA753T/G, ntC787A/G, ntA805C/T, ntA894G/C were significantly more correlated with genotype B (*P* < 0.05), while ntC/T724G, ntT820C, ntC837T, ntT840C, ntA852G, ntC873T, ntC876T were significantly more associated with genotype C (*P* < 0.05).

### Comparison of resistance mutations between treatment-naive and post-treatment patients

**Figure 4.**
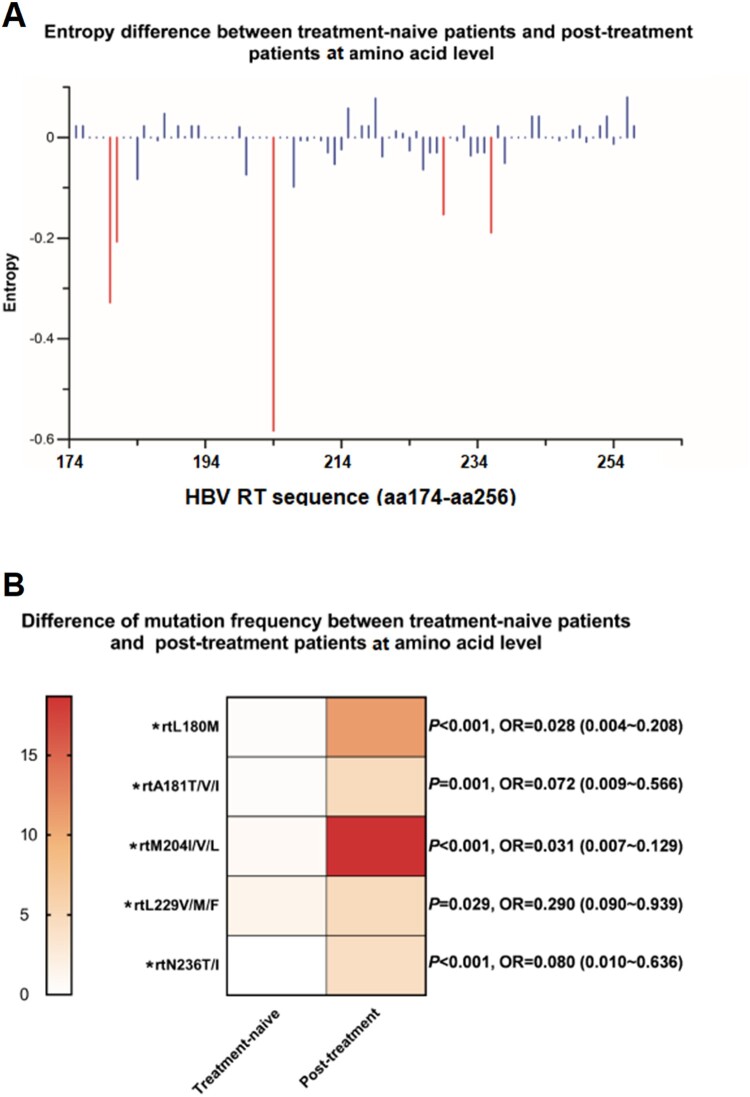
Analysis of mutations between treatment-naive patients and post-treatment patients. (A) 83 amino acid sites (rt174-rt256) within RT region were aligned and assessed for Shannon entropy of each amino acid position. Bars reflect the Shannon entropy (genetic variability) at amino acid sites. The red bars indicate entropy with significant differences via a pairwise comparison between treatment-naive and post-treatment patients. (B) 5 amino acid sites with significant differences of entropy between treatment-naive and post-treatment patients were screened, frequency of mutations at these 5 sites were determined and compared by *t*-test*P < 0.05. nt: nucleotide.

The characteristics of mutation frequency and sequence entropy at AA level between different therapy status is shown in Figure 4. As the method of entropy analysis described in the previous part, our study also revealed that a survey for the Shannon entropy might play a constructive role in identifying the hyper-variable sites which potentially correlated with an acceleration of viral resistance. In general, a narrow range of entropy (0–0.08) observed in the treatment-naive group changed largely due to the selective pressure under NAs therapy with the entropy peak up to 0.58 (at the site rt204) in post-treatment group (Figure 4(A)). Five AA sites identified with the significantly elevated entropy levels were rtL180M, rtA181T/V/I, rtM204I/V/L, rtL229V/M/F, rtN236T/I, four of which had been widely known already as classical resistance mutation [14]. rtL229 substitutions were the only putative resistance mutations that had been reported to be associated with long-term NAs use but remained to be further clarified in vivo and in vitro. We thus proceeded with an assessment of the AA mutation frequency. As a result, the frequency of rtL229V/M/F among the post-treatment populations tended to be significantly higher than that among treatment-naive ones (*P* = 0.029), which indicated a similar distribution as compared with the other four primary and secondary resistance mutations (Figure 4(B)). Therefore, it suggested that rtL229 substitution might be a resistance mutation which should be verified by phenotypic resistance assay.

To investigate the clinical profile of rtL229 substitution, we analyzed the rtL229V/M/F isolates from a cohort of 214 post-treatment patients. Table 2 displays the rtL229V/M/F isolates with the characteristics of genotype, drug usage, viral load and the virological response to drug. The results revealed a relatively higher frequency of the mutation rtL229V (5/10, 50%) than rtL229M or rtL229F, and a correlation between rt229 substitutions and AA changes at rt204 or rt180 (6/10, 60%). Most of the patients with the colocalization of rtM204V/I + rtL229 V/M/F mutations experienced significant virological breakthrough (increased HBV DNA level of 2.3∼7.9 log10 IU/ml, patients with low drug compliance excluded) and had a history of prolonged LMV exposure prior to the detection of the resistance mutations.

**Table 2.** Virological characteristics of ten post-treatment patients who presented variants of rtL229V/M/F.

P	Gen	Drug usage	HBV DNA change	Mutation
1	B	LMV	8.7^b^ →ND^n^ → 7.2^s^	rtL229V + rtL180M + rtM204V
2	B	LMV	6.6^b^ →ND^n^ → 4.0^s^	rtL229V + rtM204I
3*	B	LMV	UK^b^ →ND^n^ → 6.1^s^	rtL229V
4*	B	ETV	6.0^b^ →4.3^s^ → 3.1^n^	rtL229F + rtM204I
5	C	LMV	6.3^b^ →ND^n^ → 4.2^s^	rtL229M + rtL180M + rtM204V
6	C	ETV	6.7^b^ →3.9^s^ → ND^n^	rtL229V
7	C	ETV	7.6^b^ →5.1^s^ →ND^n^	rtL229M
8	C	LMV	7.0^b^ →ND^n^ → 7.1^s^	rtL229V + rtL180M + rtM204V
9	C	LdT	5.6^b^ →ND^n^ → 7.9^s^	rtL229F + rtM204I
10	C	LMV	5.8^b^ →3.4^n^ → 5.7^s^	rtL229M

Note: P: patients; Gen: genotype; HBV DNA change: the increase or decrease in HBV DNA level(log10 IU/ml); ^b^ Baseline HBV DNA level: HBV DNA level detected prior to the therapy; ^n^ nadir HBV DNA level: the lowest HBV DNA level detected during the therapy; ^s^ HBV DNA level at the time point of sequencing; ND: HBV DNA not detected; UK: unknown HBV DNA level; LMV: lamivudine; LdT: telbivudine; ETV: entecavir; rt: reverse transcriptase; * patients with low therapy compliance.

### In-silico prediction of HBV RT three-dimensional structure

**Figure 5.**
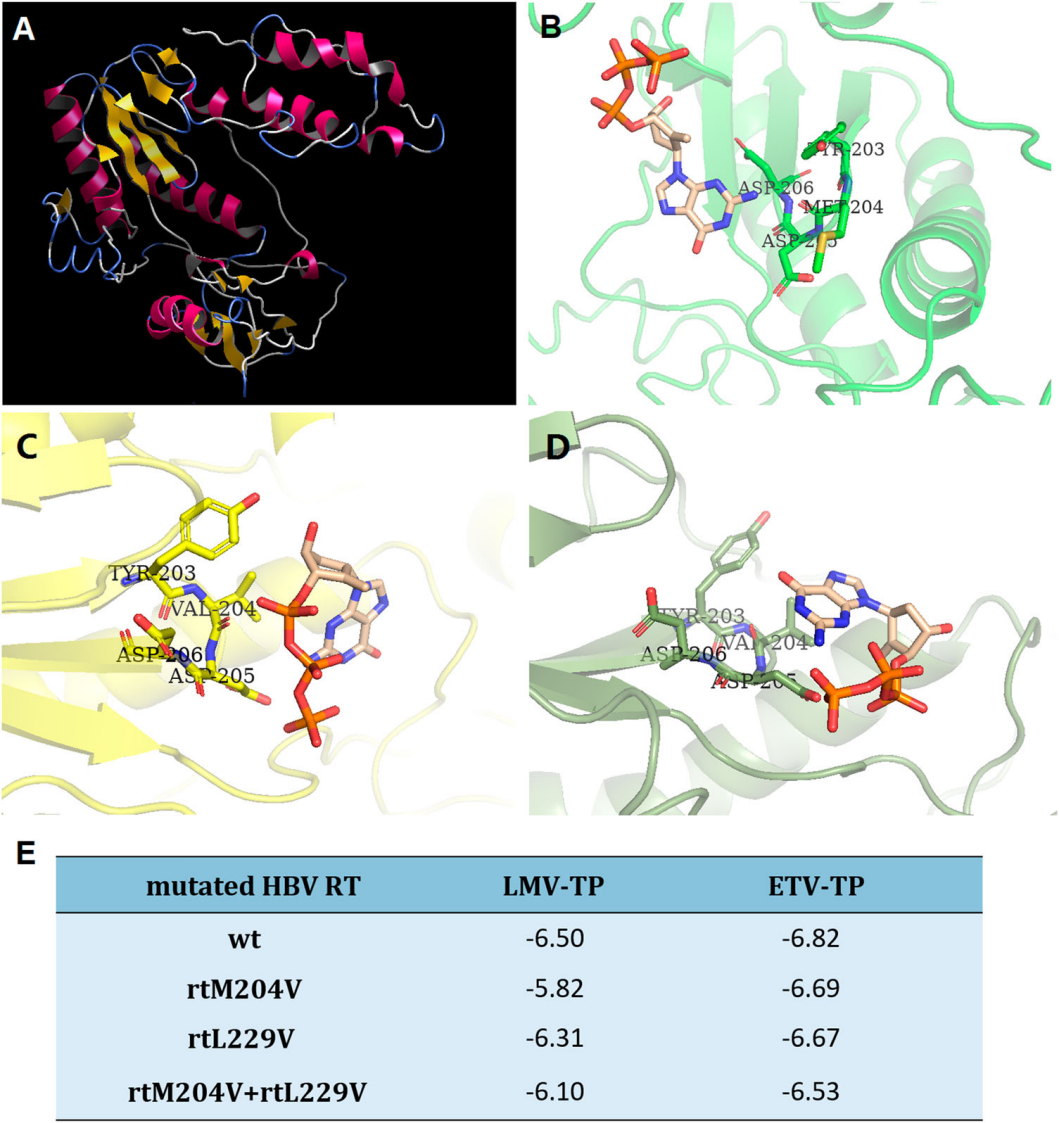
In-silico prediction of HBV RT three-dimensional structure. (A) Overall view of three-dimensional structure of HBV RT region, the structure was constructed by wild type RT sequence, and used as a receptor for further evaluation of molecular docking and calculation of binding energy. (B) Docking simulation of wild-type HBV RT to ETV-TP. (C) Docking simulation of HBV RT with rtM204V mutation to ETV-TP. (D) Docking simulation of HBV RT with rtM204V + rtl229V mutation to ETV-TP. The ETV-HBV complexes after flexible docking show the different of M204 and L229 to accommodate shifted orientation of ETV-TP in the active site of YMDD motif. (E) Binding energy (ΔG: Kcal/mol) of resistant HBV RT to NAs agents. rt: reverse transcriptase; wt: wild-type; ETV-TP: entecavir triphosphate; LMV-TP: lamivudine triphosphate. TYR: tyrosine; MET: methionine; VAL: valine; ASP: aspartic acid.

We further evaluated the effects of mutations on the binding ability of HBV RT to LMV and ETV through in-silico molecular docking simulations. Figure 5 displays the three-dimensional structure of the wild-type HBV RT and the HBV RT binding domain harboring different mutations of rtM204V and rtM204V + rtL229V. A steric hindrance between HBV RT deoxynucleoside triphosphate (dNTP) binding pocket and ETV-TP was illustrated in the molecular model of HBV RT containing rtM204V, while the introduction of rtL229V modified the conformation of side chain, thus to presumably cause an alteration of the spatial relationship between HBV RT and ETV-TP (Figure 5(A–D)). Binding energy of resistant HBV RT was calculated and compared with wild-type (Figure 5(E)). As a result, mutated RT of both rtM204V and rtM204V + rtL229V showed restricted binding affinity to LMV-TP, since binding at lower potential energy creates a more stable structure. Binding energy to ETV-TP slightly increased by HBV RT containing rtM204V, while this RT with an addition of rtL229V displayed a binding energy rise of 0.29, suggesting that the introduction of the rtL229V mutation accompanied by rtM204V might restrict ETV-TP binding site to further cause a resistance to ETV.

### Effects of rtL229V on HBV replication and antiviral susceptibility

**Figure 6.**
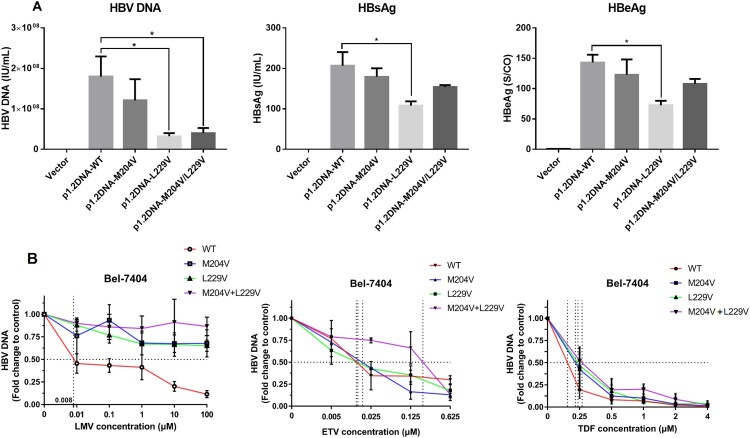
Assessment of viral replication levels and phenotypic susceptibility to antiviral drugs in vitro. (A) Bel-7404 cells were transfected with the plasmid p1.2DNA-WT, −M204V, −L229V, −M204V + L229V. HBV DNA levels, HBsAg and HBeAg of wild-type and various mutant strains were determined to evaluate the replication capacity. (B) Bel-7404 cells were transfected with the plasmid p1.2DNA-WT, −M204V, −L229V, −M204V + L229V, and subsequently treated with antiviral drugs at the indicated concentrations. HBV DNA levels of the wild-type and various mutant strains were determined after the harvest of cell culture supernatant. The 50% inhibitive concentration (IC50) of LMV, ETV, and TDF for wild-type strain and mutant strains were calculated. *Significant differences compared to wild-type. *P* < 0.05. WT: wild-type; LMV: Lamivudine; ETV: entecavir; TDF: tenoforvir; S/CO: optical density of sample/cut off value.

To assess the efficacy of rtL229V mutation, *in-vitro* assays for wild-type HBV and mutants were performed. HBsAg, HBeAg and HBV DNA in culture medium were detected after 6 days. Plasmid expression vectors harboring single mutation rtL229V, rtM204V and combination mutation rtL229V + rtM204V were constructed by site-directed mutagenesis from the wild-type plasmid. The level of HBsAg, HBeAg, and HBV DNA from Bel-7404 cells transfected with the rtL229V mutants was significantly lower than that of those with the wild-type plasmid, suggesting that the presence of the rtL229V single mutation might reduce viral replication and production of HBV antigen. The rtM204V + rtL229V mutants impaired HBV DNA at similar levels to the rtL229V single mutants, while no significance was observed in HBsAg and HBeAg decrease between the combination mutants and wild-type (Figure 6(A)).

Drug susceptibility of the mutants was tested against wild-type HBV using increasing concentrations of NAs agents (Figure 6(B)). Starting with the rtL229V single mutation, resistance to LMV began to appear and increased >1000-fold (IC_50_>10 μΜ) in contrast to wild-type (Table S4). There was no *in-vitro* resistance to ETV for either single mutation rtL229V or rtM204V, which had IC_50_ values of 0.008 and 0.020 μM (1.0-fold and 2.5-fold), respectively. The decreased susceptibility to ETV with rtL229V + rtM204V was 25.7-fold as compared with the wild-type, which could be recognized as drug resistance, but was relatively weaker than that observed with rtL229V or rtM204V-transfected cells exposed to LMV. None of the mutant strains in our study conferred resistance to TDF, suggesting a good and sustained antiviral efficacy of TDF on inhibiting viral replication. From these measurements we concluded that rtL229V mutants inhibited the antiviral susceptibility of LMV at a consistent level with the other classical LMV-resistance mutation, and further conferred resistance to ETV when accompanied by the additional rtM204V mutation simultaneously.

## Discussion

Although the wide use of NAs benefits millions of CHB patients, people remain concerned about an increasing emergence of resistance mutants constantly. The RT region displays that merely a single mutation at specific site will reduce the susceptibility to antiviral compounds, thus resulting in a virological nonresponse or drug resistance. In our study, an overall sequencing of HBV RT region was performed in treatment-naive and post-treatment patients. Only 1.1% of HBV isolates carried classical primary or secondary resistance mutation, hinting that the NA-r were mainly derived from drug selective pressure rather than natural occurrence [17–18]. It has also come to our attention that the reported incidence of pre-existing resistance mutations in treatment-naive patient was variable with the range from none to more than 5% according to different publications [8–11]. This discrepancy exists between studies correlated with the geographical or ethnic background of patients as well as viral genotype, implying a high genetic variability of HBV between various epidemic viral strains in different regions. Moreover, it was still noted that the direct-sequencing as population-based sequencing approach, might be restricted to detect major quasispecies more than 20% of the entire HBV populations, thus possibly leading to the undetection of the strains that were minor but functionally critical [19,20]. To confirm these minor strains, a high throughput sequencing detection may need to be performed.

Since specific patterns of mutations may be structurally/functionally restricted by particular genotypes, researches have increasingly focused on how HBV genotypes affect NA-r mutations within some critical motifs of RT region in CHB patients [21,22]. Through Sanger sequencing, our study assessed the influence on the types or frequency of mutations by HBV genotype, and discovered 14 genotype-dependent nucleotide polymorphic sites and 5 genotype-dependent AA polymorphic sites (Figure 1, Table S1, Table S2). In these findings, rt221, rt224, rt238 were identified to be genotype-dependent sites, as coincided with the reported literature [14,20]. rt222, rt223 were newly identified in Fujian, China and rarely reported in any other areas of China, strongly suggesting that geographical distribution of HBV genotypes would also be a contributing factor in HBV genome sequence discrepancies. Moreover, our results highlight that identification of the genotype-dependent sites would be important for the distinction between mutations and mere polymorphisms during mutation analysis, for example, glutamine at rt238 as a low-frequency AA residue in our study was more likely to be a naturally occurring mutation rather than a polymorphism site. In addition, the standard for differentiating genotypes by our genotype-dependent sites analysis may only validate in genotype B and C samples, considering that other viral genotype, like genotype D may display a completely different characteristic of genotype-dependent sites [23,24].

How HBV genotypes potentially modulate NAs resistant mutation still remains controversial [14,25,26]. A recent study published by Li et al. (2017) [27] revealed that genotypes B and C preferentially utilized different mutations of LMV resistance, clarifying a higher risk for C-genotype HBV to develop multidrug-resistance mutations. A similar view was also proposed in a cohort of genotype A and D HBV-infected patients [28]. Both of the findings showed a possible role for HBV genotypes in driving the evolution of NA-r mutation. In our study, genotype B and C were compared regarding the number of isolates with mutation within RT region. Followed by the further investigation of some given sites, we found substitutions at two NA-r mutation sites rtV191I and rtA181T/V, were of interest due to detection much more frequently in genotype C than genotype B (Figure 3(C), Figure 3(D)), indicating different genomic variability by each genotype. rtV191I as a putative resistance mutation, was the only mutation with genotype-specificity identified in the treatment-naive patients. Of particular concern, the potential effects of rtV191I might result in decreased viral replication, impaired secretion of HBsAg and the LMV, ADV related resistance [14,29]. However, the effects of genotype might not be considered as the main factor modulating the resistance evolution in our study, owing to a finite number of genotype-specific NA-r mutations as well as the discovery of the synonymous mutations which comprised the majority of genotype-specific mutations. Some of these synonymous mutations were found with a notably high frequency among the respective genotype (e.g. ntG753T,>40% among genotype B), which might play a role in the existence of genotype-dependent nucleotide polymorphic sites, whereas most of them actually tended to have no pathogenic efficacy in phenotype.

Our study also paid attention to the evolutionary pattern of resistance mutants as a result of antiviral therapy. We calculated Shannon entropy that referred to a quantitative measurement of uncertainty in a collection of AA or nucleotide sequences. Higher entropy indicates various types of AAs or nucleotides easily occupy the specific site without predominant ones, thus reflecting the genetic background involving potential resistance mutations [30,31]. Generally, in this study, there were five AA substitutions associated with NAs treatment, four of which had been confirmed as primary and secondary resistance mutations (Figure 4(B)). Of these mutations, rtL180M and rtM204V/I were predominant in both genotype B and C, and probably favored by genotype D according to another report in Italian people, supporting that rtL180M and rtM204V/I could be joint resistance mutations across most genotypes of HBV [28]. Notably, NAs treatment not only contributed to a major selective factor for the primary and secondary NAr mutations, but also induced the changes at those sites with unconfirmed resistance to NAs. Concretely, we have identified putative resistance mutations rtL229V/M/F whose frequency significantly increased in treated patients. The characteristic summarized here indicated that rtL229 mutations cluster with rtM204 mutations with relatively high frequency (in 6 of 10 patients, Table 2) when compared with a previous report [23]. Furthermore, the significant difference of the prevalence of rtL229V/M/F mutations between treatment-naive and NAs-treated patients was consistent with that observed from rtM204V, indicating that this mutation may contribute to the antiviral resistance.

It was described in several reports that rtL229 mutants, mainly involving rtL229F and rtL229W, conferred resistance to LMV and showed a capacity of restored viral replication in vitro when coexisted with rtM204 V/I [32,33]. However, the rtL229V, which accounts for the majority of rtL229 substitutions in Chinese people, was not analyzed in detail in these papers [33]. Our *in-vitro* testing results revealed that the rtL229V and rtL229V + rtM204V produced the most potent suppression of viral replication, followed by rtM204V single mutation (Figure 6(A)). From earlier reports, YMDD motif mutants tended to replicate in vitro with a lower efficiency compared to that of wild-type HBV, due to HBV polymerases’ lower affinities arisen from the active catalytic site alteration than wild-type polymerases for the natural dNTP substrates [34–36]. Our data partly confirmed these findings with even more significant replication inhibition in rtL229V mutants compared to rtM204V mutants, and argued against the observations of enhanced viral replication through adaptive changes. These properties interestingly corroborated with those of ETV resistance mutations rtT184G/rtS202I in a study of the mechanistic basis underlying ETV resistance [37,38]. Taking into account the comprehensive analysis of both phenotypic and molecular modeling we have described here (Figure 5), it was reasonable to hypothesize that the highly decreased viral fitness and binding affinity restriction of the rtL229V substitution were much more similar to the ETV-resistant mutation, rather than other substitutions at rt229, such as rtL229F or rtL229W. It is noteworthy that the catalytic efficiency of the ETV-resistant polymerases was reduced to approximately 2–3 fold lower than wild-type as proposed by Walsh et al 2010 which confirmed this mechanism as a basis for impaired HBV replication [37]. Determining whether a similar phenomenon is observed in rtL229 V mutants may call for a further enzyme kinetic study.

In phenotypic resistance testing, rtL229V single mutation showed a >1000-fold increased IC_50_ value for LMV, in line with the results from the rtM204V phenotypic testing and the clinical profile of patients shown in Table 2. Of note, the IC_50_ increasing by a factor of over 25-fold was identified as a decrease in ETV susceptibility. These results demonstrated that rtL229V displayed a negative efficacy of ETV compensatory mutation, and could confer as much resistance to ETV as rtT184G/rtS202I/rtM250V mutants [39,40]. The introduction of rtL229V into HBV RT might confer resistance through the mechanism of restricting the size of the ETV-TP binding pocket to sterically impede the ETV-TP binding, and presumably interact with rtM204V through steric repositioning of the YMDD loop [40]. In addition, considering that rtL229 substitutions emerged and displayed significant virological breakthrough in 83.3% (5/6) of the cases who have received LMV only (Table 2), it suggested that the reduced ETV susceptibility from rtL229V might be easily developed together with or subsequent to the emergence of rtM204V among the cases of LMV therapy failure. Therefore, the sequential treatment with LMV and ETV should be avoided, while an early diagnosis of rtL229V which was supported as important clinical marker of ETV resistance were recommended, as far as our study was concerned.

In conclusion, our study involved important mutational characteristics of treatment-naive and post-treatment Chinese patients with HBV infection by Sanger sequencing. The identification of genotype-dependent polymorphic sites and genotype-specific resistance mutations demonstrated HBV genetic variability to some degree between genotype B and C, suggesting a limited role for HBV genotype in driving the resistance change during the drug usage. rtL229V as putative resistance mutation, was critically selected by drug pressure and showed an increased IC_50_ value for ETV in vitro when coexisting with LMV resistance mutation rtM204V, suggesting that rtL229V had a negative efficacy of ETV mutation, in agreement with the discovery from our analysis of sequencing, clinical data and molecular modeling. Our findings provide novel insights into the evolution of antiviral resistance, thus to provide the basis for a rational administration of antiviral therapy.

## Supplementary Material

Supplementary_material.docx
