# Supplementary material for: Mutational characterization of HBV reverse transcriptase gene and the genotype-phenotype correlation of antiviral resistance among Chinese chronic hepatitis B patients
Source: Emerg Microbes Infect. 2020 Oct 30;9(1):2381–93. doi: 10.1080/22221751.2020.1835446 (PMC7605321; doi:10.1080/22221751.2020.1835446)
Supplement: Supplementary_material.docx [file TEMI_A_1835446_SM6169.docx]

| **Genotype** | **nt667** | | | | | | | | | |  | | **nt724** | | | | | | | | | | | | |  | **nt791** | | | | | | |  | **nt793** | | | | | | |
| --- | --- | --- | --- | --- | --- | --- | --- | --- | --- | --- | --- | --- | --- | --- | --- | --- | --- | --- | --- | --- | --- | --- | --- | --- | --- | --- | --- | --- | --- | --- | --- | --- | --- | --- | --- | --- | --- | --- | --- | --- | --- |
|  | T | | C | | | A | | | ***P*** | |  | | C | | | T | | G | | | | ***P*** | | | |  | A | | T | | | ***P*** | |  | G | | A | | ***P*** | | |
| **B** | 160 | | 2 | | | 0 | | | <0.01 | |  | | 161 | | | 1 | | 0 | | | | <0.01 | | | |  | 162 | | 0 | | | <0.01 | |  | 126 | | 36 | | <0.01 | | |
| **C** | 1 | | 121 | | | 1 | | |  |  |  | | 3 | | | 116 | | 4 | | | |  |  |  |  |  | 6 | | 117 | | |  |  |  | 3 | | 120 | |  |  |  |
|  | **nt796** | | | | | | | | |  | | **nt799** | | | | | | | | | | | |  | **nt834** | | | | | | |  | **nt841** | | | | | | | | |
|  | G | | T | | | A | | ***P*** | |  | | G | | | A | | | C | | | ***P*** | | |  | A | | | G | | ***P*** | |  | C | | A | | G | | | ***P*** | |
| **B** | 162 | | 0 | | | 0 | | <0.01 | |  | | 159 | | | 0 | | | 3 | | <0.01 | | | |  | 161 | | | 1 | | <0.01 | |  | 162 | | 0 | | 0 | | | <0.01 | |
| **C** | 15 | | 107 | | | 1 | |  |  |  | | 14 | | | 109 | | | 0 | |  |  |  |  |  | 41 | | | 82 | |  |  |  | 6 | | 116 | | 1 | | |  |  |
|  | **nt843** | | | | | | | | | | | | | | | |  | **nt849** | | | | | | | | | | | | | | | |  | **nt853** | | | | | | |
|  | C | | | T | | | G | | | A | | | | ***P*** | | |  | A | | | | C | | | | | T | | G | | | ***P*** | |  | A | | C | | | ***P*** | |
| **B** | 158 | | | 3 | | | 0 | | | 1 | | | | <0.01 | | |  | 156 | | | | 3 | | | | | 0 | | 3 | | | <0.01 | |  | 154 | | 8 | | | <0.01 | |
| **C** | 4 | | | 118 | | | 1 | | | 0 | | | |  |  |  |  | 5 | | | | 115 | | | | | 3 | | 0 | | |  |  |  | 1 | | 122 | | |  |  |
|  | | **nt855** | | | | | | | | | | | | | | | |  | **nt861** | | | | | | | | | | | | | | | |  | **nt864** | | | | | |
|  |  | A | | | T | | | | C | | | | G | | | ***P*** | |  | A | | | | T | | | | | C | | | G | | ***P*** | |  | T | | C | | | ***P*** |
| **B** | | 158 | | | 1 | | | | 0 | | | | 3 | | | <0.01 | |  | 157 | | | | 1 | | | | | 2 | | | 2 | | <0.01 | |  | 161 | | 1 | | | <0.01 |
| **C** | | 3 | | | 118 | | | | 1 | | | | 1 | | |  |  |  | 3 | | | | 118 | | | | | 1 | | | 1 | |  |  |  | 10 | | 113 | | |  |

**Table S1.** Genotype-dependent nucleotide polymorphic sites in the reverse transcriptase gene of hepatitis B virus from samples of treatment-naive patients.

**Note**: n=162 genotype B, n=123 genotype C; A: adenine; T: thymine; G: guanine; C: cytosine; nt: nucleotide.

**Table S2.** Genotype-dependent amino acid polymorphic sites in the reverse transcriptase region of hepatitis B virus from samples of treatment-naive patients.

**Note:** n=162 genotype B, n=123 genotype C; Y: tyrosine; F: phenylalanine; H: histidine; A: alanine; T: threonine; S: serine; V: valine; I: isoleucine; L: leucine; N: asparagine; Q: glutamine; rt: reverse transcriptase; ***^a^*** Described as naturally occurring mutations in this

| **Genotype** | **rt221** | | | |  | **rt222** | | |  | **rt223** | | | |
| --- | --- | --- | --- | --- | --- | --- | --- | --- | --- | --- | --- | --- | --- |
|  | Y | F | H ***^a^*** | ***P*** |  | A | T | ***P*** |  | A | S | T ***^a^*** | ***P*** |
| **B** | 160 | 0 | 2 | <0.01 |  | 126 | 36 | <0.01 |  | 162 | 0 | 0 | <0.01 |
| **C** | 6 | 117 | 0 |  |  | 3 | 120 |  |  | 15 | 107 | 1 |  |

|  | **rt224** | | | | |  | **rt238** | | | | | |
| --- | --- | --- | --- | --- | --- | --- | --- | --- | --- | --- | --- | --- |
|  | V | I | L ***^a^*** | T ***^a^*** | ***P*** |  | H | N | Q ***^a^*** | A ***^a^*** | S ***^a^*** | ***P*** |
| **B** | 159 | 0 | 3 | 0 | <0.01 |  | 161 | 0 | 1 | 0 | 0 | <0.01 |
| **C** | 14 | 107 | 0 | 2 |  |  | 6 | 115 | 0 | 1 | 1 |  |

**Table S3. Prevalence of the potential NA-r Mutations identified by sequencing analysis**

| **Mutation category** | **Mutation types** | **Treatment-naive**  **(n=285)(%)** | **Post-treatment**  **(n=214)(%)** | ***P*-value** |
| --- | --- | --- | --- | --- |
| 1. Primary NA-r mutation | rtA181V/T/I  rtT184S/L  rtM204V/I  rtN236T/I | 1 (0.4)  0 (0.0)  2 (0.7)  0 (0.0) | 10 (4.6)  3 (1.4)  32 (15.0)  8 (3.7) | 0.001  0.072  <0.001  <0.001 |
| 2 Secondary/compensatory NA-r mutation | rtL180M | 1 (0.4) | 24 (11.2) | <0.001 |
| 3. Putative NA-r mutation | rtV191I  rtA200V  rtV207I  rtS213T  rtV214A  rtE218D  rtL229V/M/F  rtI233V  rtH/N238D/Q/S/A/K/R*  rtS/C256G | 5 (1.8)  0 (0.0)  0 (0.0)  13 (4.6)  3 (1.1)  1 (0.4)  2 (0.7)  1 (0.4)  1 (0.4)  2 (0.7) | 3 (1.4)  3 (1.4)  3 (1.4)  12 (5.6）  2 (0.9)  0 (0.0)  10 (4.7)  1 (0.5)  2 (0.9)  3 (1.4) | 0.999  0.076  0.178  0.537  0.700  0.999  0.029  0.576  0.462  0.656 |
| 4.Pre-treatment mutation | rtV/I224L/T* | 14 (4.9) | 12 (5.6) | 0.999 |

**Note:** rt: reverse transcriptase; NA-r: NAs resistance mutation; *genotype-dependent AA polymorphic position identified in this study

**Table S4. Drug susceptibility analysis of HBV strains.**

| **mutated HBV strain** | **Lamivudine** | | **Entecavir** | | **Tenofovir** | |
| --- | --- | --- | --- | --- | --- | --- |
|  | **IC_50_(μM) fold** | | **IC50(μM) fold** | | **IC50(μM) fold** | |
| Wild type | 0.008 | 1.0 | 0.008 | 1.0 | 0.19 | 1.0 |
| rtM204V | >10.000 | >1000.0 | 0.020 | 2.5 | 0.23 | 1.2 |
| rtL229V | >10.000 | >1000.0 | 0.008 | 1.0 | 0.24 | 1.3 |
| rtM204V+rtL229V | >10.000 | >1000.0 | 0.206 | 25.7 | 0.26 | 1.4 |

Note: rt: reverse transcriptase. IC_50_: the 50% inhibitive concentration of drug. Fold: the IC_50_ of mutant/the IC_50_ of wild type.
